# Improving laboratory turnaround times in clinical settings: A systematic review of the impact of lean methodology application

**DOI:** 10.1371/journal.pone.0312033

**Published:** 2024-10-17

**Authors:** Negesse Cherie, Dereje Mengesha Berta, Mebratu Tamir, Zufan Yiheyis, Abiy Ayele Angelo, Amare Mekuanint Tarekegn, Elias Chane, Mesele Nigus, Bisrat Birke Teketelew

**Affiliations:** 1 Department of Quality Assurance and Laboratory Management, School of Biomedical and Laboratory Science, College of Medicine and Health Science, University of Gondar, Gondar, Ethiopia; 2 Department of Hematology and Immunohematology, School of Biomedical and Laboratory Science, College of Medicine and Health Science, University of Gondar, Gondar, Ethiopia; 3 Department of Medical Parasitology, School of Biomedical and Laboratory Science, College of Medicine and Health Science, University of Gondar, Gondar, Ethiopia; 4 Department of Immunology and Molecular Biology, School of Biomedical and Laboratory Science, College of Medicine and Health Science, University of Gondar, Gondar, Ethiopia; 5 Department of Clinical Chemistry, School of Biomedical and Laboratory Science, College of Medicine and Health Science, University of Gondar, Gondar, Ethiopia; Hawassa University College of Medicine and Health Sciences, ETHIOPIA

## Abstract

**Background:**

Lean methodology, originally developed in the manufacturing sector, is a process management philosophy focused on maximizing value by eliminating waste. Its application in laboratory settings, particularly concerning laboratory turnaround times (TAT), involves a systematic approach to identifying inefficiencies and optimizing processes to enhance value for end customers.

**Methods:**

This systematic review was registered in PROSPERO with identification number (CRD42024552350) and reported based on the 2020 PRISMA checklist. An extensive search strategy was performed using PubMed, Scopus, and Embase databases and gray literatures. Advanced searching was used using Boolean operators (AND & OR). After articles were exported to endnote x8, duplications were removed and articles were selected based on titles, abstracts, and full texts. The illegibility of the articles was independently assessed by the three authors (NC, DMB, and BBT), and the disagreements were settled through scientific consensus. Methodological quality was assessed using JBI critical appraisal checklist.

**Discussion:**

In this review, electronic databases search yielded 1261 articles, of which 7 met the inclusion criteria. The review demonstrated, implementation of lean principle into the routine laboratory testing had an overall impact 76.1% on reducing laboratory TAT. Transportation, manual data processing, inefficient workflow, and the heavy workload were identified as the main wasteful procedures. To eliminate these non-value-added steps, several intervention techniques were implemented, including the use of a barcoding system, process redesign, workflow optimization, hiring additional staff, and relocating the sample collection room closer to the result distribution center. Lean implementation is crucial in the medical laboratory industry for optimizing processes, reducing TAT, and ultimately enhancing customer satisfaction. As a result, all clinical laboratories should adopt and implement lean principles in their routine testing processes. The medical laboratory industry should also proactively look for and apply lean tools, provide ongoing training, and foster awareness among laboratory staffs.

## Introduction

Lean methodology is a process management philosophy [1], developed in the business industry by Toyota executives [2], that examines organizational processes to limit the use of resources for those processes that provide value for the end customer. Lean was introduced into the healthcare sector in the late 1990s and has grown continuously across the world [3, 4]. Many hospitals have adopted the lean thinking approach in areas like reducing registration time [5], cycle time in outpatient department [6], clinical laboratory [7], emergency care services [8], and nurses scheduling [9] proving its potential to improve both the efficiency and quality of health care delivery [10].

Standard operating procedures, root cause analysis, downtime and performance, key performance indicators, visual management (warning and regulatory signs), Kaizen (continuous improvement), 5S (sort, straighten, shine, standardize, sustain), PDCA (Plan Do Check Act) or Deming cycle, Kanban (Inventory Regulator), and value stream mapping (VSM) are some of the lean tools that are used in the medical laboratory industry [11]. However, VSM is the most common lean methodology for examining the existing situation and planning the course of events from specimen receipt to result reporting [12]. It is the first step towards lean implementation and observes where the value is created and highlights the waste [13]. This powerful tool not only highlights process inefficiencies, transactional, and communication mismatches but also guides about future improvement [14].

In the VSM process, activities are classified into value-adding (VA) and non-value-adding (NVA) categories. Value-adding activities are those that enhance the product or service in a way that the customer perceives as valuable and is willing to pay for. These activities directly contribute to the final product or service. Non-value-adding activities, on the other hand, do not directly contribute to the final product or service from the customer’s perspective. They often involve waste or inefficiency, which can lead to increased costs and reduced productivity [13]. Lean identifies seven types of wastes in the services such as: waiting, over production, over processing, inventories, transportation, motion and defects [15] to which eighth waste of underutilization of skills has also been added [13].

The principle behind lean involves leveraging human resources and available tools to enhance user services. Implementing the Lean methodology, particularly in healthcare settings such as clinical laboratories, can significantly improve workflow and performance [16–18]. Integrating Lean improvement models into the total testing process has notably impacted the laboratory turnaround time for delivering clinical test results [19]. Lean methodologies focus on reducing waste, optimizing processes, and fostering a culture of continuous improvement, leading to more efficient and effective healthcare delivery. This approach ensures that clinical laboratories can meet the increasing demands for timely and accurate test results, ultimately enhancing patient care and operational efficiency. The implementation of lean principles had an impact on the quality improvements in 85% of the clinical laboratory industry by improving operational performance, reducing turnaround time, and improving customer satisfaction [11].

### Laboratory Turnaround Time (TAT)

Turnaround time in a clinical laboratory is the amount of time that passes between the time in which the test is requested and the laboratory result being reported [20, 21]. However, the definition of TAT might change based on where the cycle begins, such as ordering tests, phlebotomy, and receiving results from the laboratory. It can also be categorized by specific stages of the process, including pre-analytical (order to preparation), analytical (sample received to result released), and post-analytical (reporting to action), or by request priorities (STAT, urgent, and routine) [22].

Turnaround time is an important performance parameter in clinical laboratory services which faster’s delivery of services as one of the goals for quality assurance. Delay in patient diagnosis adversely affects patient outcomes [23]. Thus, the technicians prefer a reduced TAT of the tests, which helps to diagnose, treat and discharge their patient earlier, leading to patient satisfaction and improved efficiency of clinicians [24].

Nowadays, significant importance is given to the patient satisfaction hence laboratories aim at enhanced delivery through reduced TAT [25]. Thereupon, dissatisfaction on test TAT by clinicians has become a major source of complaints to the laboratory’s service. Consequently this led to consumption of much time and effort by the laboratory in order to resolve the complaint and improve the service [26]. Furthermore, increased laboratory TAT not only reduces the overall quality of care and optimal use of human resources but also increases the financial burden on patients and leads to decreased customer satisfaction [27, 28]. About 75% of the total test TAT is contributed by pre- and postanalytical phases activities [29].

Lean methodology effectively reduces turnaround times (TAT), eliminates waste, and directly enhances patient satisfaction with laboratory processes and reports. Additionally, it empowers workers [30]. By adopting Lean principles, healthcare professionals can improve their work processes and identify opportunities to save time, money, materials, supplies, and goodwill. The long-term results of implementing Lean methodology include significant improvements in speed, quality, profitability, and customer satisfaction. This review aimed to ascertain how using Lean methodology shortens laboratory TAT and increases patient satisfaction. Additionally, it underscores the significance of integrating Lean principles into laboratory operations for ongoing quality enhancement and improved patient care outcomes.

## Materials and methods

### Protocol and registration

This systematic review was conducted to compile the most recent pieces of evidence using published and gray literature on the impact of lean implementation on laboratory TAT. The protocol for this review was registered on the Prospective Register of Systematic Reviews (PROSPERO) international database (protocol registration number: CRD42024552350). This review followed the protocol of the Preferred Reporting Items for Systematic Review and Meta-Analysis (PRISMA) guidelines [31].

### Eligibility criteria

Institutional-based studies, observational and experimental studies, articles published in English language, studies conducted on the application of lean, and published until May 2024, were included. Whereas, review papers, case reports, abstracts without full-length articles, full-length articles that did not report the outcome of interest, and non-English articles were excluded.

### Information source and search strategies

Extensive search strategies were conducted using distinct electronic databases; such as PubMed, Scopus, and Embase and gray literatures. To find studies that were missed by electronic database searches, further manual searches were conducted using online higher education repositories, government institutes, and reference lists. Keywords such as lean, laboratory TAT, and clinical laboratory were served as the basis for the search terms. Using the Boolean operator "AND," the keywords "lean," "turnaround time," and "clinical laboratory services" were combined and their synonyms were combined using the "OR" Boolean operator. Appropriate entry and search terms were used by combining the Boolean operators; ((((((((lean [Text Word]) OR ("lean methodology"[Text Word])) OR ("lean principle"[Text Word])) OR ("Toyota production system"[Text Word])) AND ("Laboratory turnaround time"[Text Word])) OR ("Laboratory TAT"[Text Word])) OR ("turnaround time"[Text Word])) OR (TAT [Text Word])) AND ("Clinical laboratory"[Text Word]). The search was restricted to studies with full text, published in the English language **(S1 Table).**

### Study selection

The studies identified through different database searches were exported to Endnote X8.1 software, and duplicated studies were removed. The selected articles were independently screened by the authors for relevance to the review objective based on the titles and abstracts. After the screening of titles and abstracts, the full texts of articles considered relevant were retrieved. The eligibility of each article was assessed independently by three authors (NC, BBT, and DMB) after reviewing the full texts. The disagreements between the authors were settled through scientific consensus.

### Data extraction and management

After careful assessment of the quality and critical appraisal of the studies, data elements were subjected to data extraction using a Microsoft Excel spreadsheet. For each article that met the eligibility criteria, the first author’s name, year of publication, study design, study area, laboratory department, laboratory tests, sample size before and after the intervention, TAT before lean, TAT after lean, changes in TAT, wasteful steps identified, and intervention measures taken were extracted **(S2 Table).** Missing data was handled according to its impact on the outcome of interest: if the missing data affected the outcome (TAT before and after lean), the article was excluded; if the missing data did not affect the outcome (publication year, study area, study design, sample size,), the remaining data was collected and the article was included in the review. But their methodological quality was assessed using JBI critical appraisal checklist.

### Assessment of the methodological quality of the studies

The Johanna Briggs Institute (JBI) checklist of related items, sampling, eligibility protocols, description of study subject and setting, appropriate statistical analysis, case definition, confounder identification, valid and reliable result measurement, bias minimization, comparability among study participants, and generalizability of the study were checked independently. The scoring system was No (not done), Yes (done), UC (unclear), and NA (not applicable), and the judgments of the score range for cross-sectional studies were 0 (lowest quality) to 8 (highest quality) and for quasi-experimental studies, 0 (lowest quality) to 9 (highest quality) [32, 33]. Articles with an average methodological score of ≥50% for each item were included in this review (**S3 Table).**

### Data synthesis

Data were extracted using a Microsoft Excel 2021 spreadsheet. A qualitative synthesis of publications has been performed and the percentage of TAT reduction for each article was calculated by dividing the change in TAT to the TAT before lean and multiplied by 100 (i.e., %TAT reduction = change in TAT/TAT before lean*100). The overall TAT reduction percentage was calculated by dividing the sum of change in TAT to the sum of TAT before lean and multiplied by 100 (i.e., Overall % of TAT reduced = ∑change in TAT/∑TAT before lean*100). Finally, results were reported using text, tables, and figures.

### Outcome of interest

The outcome of interest was the impact of lean implementation on laboratory TAT.

## Results

### Study selection

**Fig 1:**
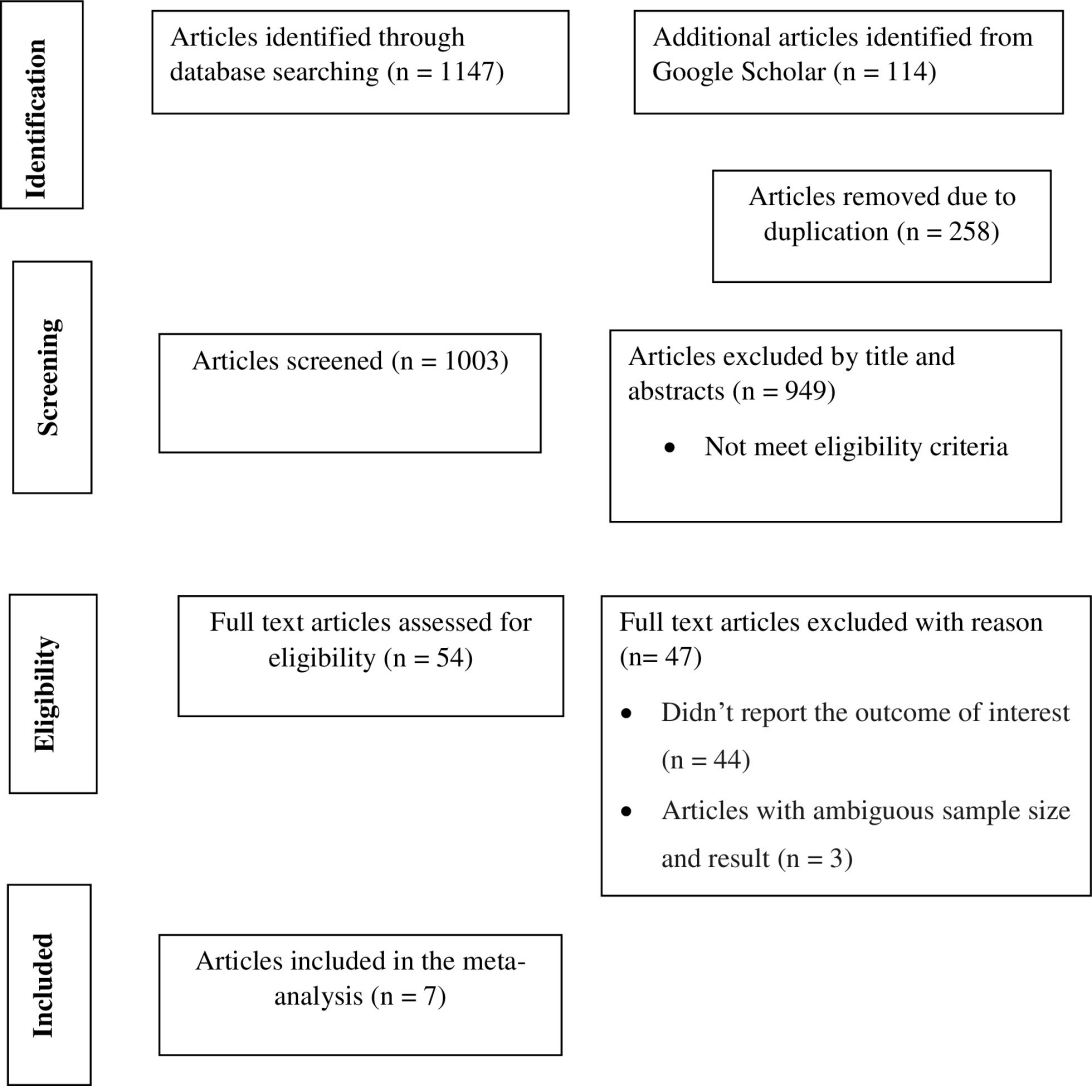
PRISMA flow chart describing screening protocols of studies for systematic review.

Initially, 1261 articles were retrieved through an extensive search of electronic databases and other gray literature. From these, 258 articles were removed due to duplication. Then, approximately 1003 records were screened for titles and abstracts. After careful assessment, 949 articles were discarded by title and abstract. Moreover, 54 full-length records were assessed for eligibility. Finally, 7 articles met the inclusion criteria **(Fig 1).** A total of 47 studies were excluded from the study: 44 didn’t report the outcome of interest, and 3 had ambiguous results.

### Study characteristics

A total of 535,831 laboratory tests (255,199 Before lean and 280,632 After lean) were evaluated for their change in TAT in the 7 included studies. Studies were conducted to assess the effect of the implementation of lean methodology in the clinical laboratory efficiency by evaluating the change in laboratory TAT. The majority of the studies were conducted in the pathology laboratory [10, 13, 34, 35], two within the emergency department [19, 36], and one in the microbiology laboratory [37]. Moreover, two studies were interventional studies [13, 35], two cross-sectional [10, 34], two longitudinal [36, 37] and one quasi-experimental [19].

### Results of individual studies

In this review, a total of seven original articles were included. Majority of the studies were conducted in the pathology laboratory, with two in the emergency setting and one in a microbiology laboratory. Three of the studies examined the overall testing process, while the remaining studies focused on a specific section of the laboratory. Below is the summary of studies included in the review, highlighting time improvements, non-value-added steps, and the intervention measures taken **(Table 1).**

**Table 1:** Non-value-added activities identified and their intervention measures through the implementation of lean methodology as extracted from different literatures.

Authors	TAT before lean	TAT After lean	Total TAT reduced	NVA steps identified	Intervention measures taken
YR Mujtabai et al., [10]	8,022 minutes	1,885 minutes	6,137 minutes	• Transportation • Processing time • Delayed accessioning • Next day grossing, and • Pending microscopic examination and final reporting	• Making streamline flow • Decreasing technologist travel events • Dealing one sample at a time • Immediate processing of the sample • Immediate collection of samples and grossed on the same day • Maintain ideal processing time • Immediate transcription of results after examination • Create awareness for the staffs about lean.
S Isa et al., [34]	35 minutes	31 minutes	4 minutes	• Repetition of task during test entry and demography • Low utilization of employee’s ability and capacity, and • Serial tracing of results by MLTs	• Using batch reduction • Visual signal • Work process standardization and workflow modification • Educating the staffs on the importance of Lean management • Appointment of STAT MLTs team members
Roe Rutledge & Joame Simpson [35]	54 minutes	23 minutes	31 minutes	• Specimen flow for transport • Technologists walk patterns in the laboratory for phone calls, dilution, sample problems, and restocking	• Duplicated backup instruments were incorporated • Activities interrupted the technologist, were handled by a person outside the cell
Shradha Gupta, Sahi Kapil, and Monica Sharma [13]	447.2 minutes	302.7 minutes	144.5 minutes	• Technicians are often busy in doing clerical works • Specimens coming with phone calls from different departments • Patient travel time to collect the results • Manual data collection	• Appointment of a dedicated staff for doing the clerical work • Shift the specimen collection room along with report delivery centres • Implementation of an automated bar-coding system to collect data • Assign part time staff for calls
Benjamin A. et al., [36]	338 minutes	186 minutes	152 minutes	• Sample processing by nurse • Sample transportation • Lab technician travel time to collect samples from ED	• Focusing the technologists’ effort on testing • Colocalization of screening and reflex testing devices • Moving testing devices closer to the sample delivery tube system, and • Leveraging existing automated testing platforms
Letelier P et al., [19]	138 minutes	126 minutes	12 minutes	• Lab staff functions • Workflow of the procedure • Infrastructure of the lab	• Process redesign • Redistributions of laboratory staff functions, and Prioritization of activities
Acero R et al., [37]	2,626 minutes	755 minutes	1,871 minutes	• Manual sample registration, identification, separation, re-racking, and tube uncapping • Manual sample transports and unnecessary staff movements	• Elimination of the 24 h storage in the refrigerator • Process workflow optimization • Automation implementation.

**Key:** TAT: Turnaround time; ED: Emergency Department; TT: Troponin Total; US: Urine Sedimentation; TI: Troponin I; UA: Urinalysis; UHCG: Urine Human Cortico Gonadotropic Hormone; MRT: Microbiology Rapid Testing; NVA: Non-Value Added; MLTS: Medical Laboratory Staffs

## Discussion

Value stream mapping is the most commonly used lean method among the studies included in this review. This may be because VSM is the simplest method for visually presenting value-added and non-value-added steps throughout the entire testing process. Additionally, it facilitates understanding how each step and processes is interrelated and integrates easily with other lean methodologies and techniques [38]. According to the findings of this review, through the application of lean, the total TAT was reduced from 11,660.2 to 3308.7 minutes, with a 71.6% reduction. This indicates that a total of 8,351.5 non-value-added minutes were removed from the routine laboratory operation through lean application, which reduced the patient waiting time and further increases patient waiting times.

A study conducted in Lahore on gastrointestinal (GI) biopsies showed that the laboratory TAT was reduced from 8022 minutes to 1885 minutes, indicating 76.5% improvement and that the process efficiency increased from 17.4% to 72.94% [10]. Transportation, processing time, delayed accessioning, next day grossing, and pending microscopic examination and final reporting were the major wasteful steps identified. Based on the findings of the study, making streamline [39], decreasing technologist travel events [40], dealing with one sample at a time [41], immediate processing of the sample [42], immediate collection of samples and gross on the same day, maintaining ideal processing time, immediate transcription of, and creating awareness for the staff about lean were the intervention measures taken. These interventions uncover the positive impact of lean on process efficiency, customer satisfaction, and continuous quality improvement by reducing laboratory TAT.

A retrospective study reported on an urgent test laboratory in Malaysia showed that the total TAT for the renal profile test was reduced from 35 minutes to 31 minutes, 11.4% reduction. Repetition of tasks, low utilization of employee’s ability and capacity, and serial tracing of results were wastes related to extra processing and increased waiting times. The major intervention measures were batch reduction, work process standardization, and workflow modification which remove four non-value-added minutes and increase process efficiency from 82.8% to 93.5% [34]. This is because working with small batch sizes has tremendous impact to improve workflow and lets us deliver quickly and reach process completion earlier.

According to the study conducted at the University of Washington School of Medicine in the core laboratory operations, the adoption of lean had reduced mean TAT of the creatinine test, from 54 minutes to 23 minutes with 57.4% reduction. The major wasteful steps identified were specimen flow for transport and technologists walk patterns in the laboratory. As a result, incorporation of duplicate backup instruments and the handling of the activities that interrupted the technologist which remove 31 non-value-added minutes resulting in a 20% improvement in the process efficiency and productivity of laboratory staff [35]. This reduces laboratory TAT because the backup instrument eliminates the time wasted during machine failure and downtime. On the other hand, handling the other activities enables the laboratory technologist to be focused on the laboratory testing.

A study conducted in the USA pathology laboratory of hematology and biochemistry reported that, after the implementation of lean, the total TAT in hematology reduced from 179.49 to 94.7 minutes, which indicate a reduction of 47.2% and from 267.71 to 208 minutes for biochemistry with 22.3% reduction. According to the findings of the study, workload, specimens come with phone calls from different departments, patient travel time to collect the results, and manual data processing were wasteful steps identified [13]. Appointment of dedicated and part-time staff for the clerical work and phone calls, shifting the specimen collection room along with report delivery centers, and the implementation of an automated bar-coding system were the major interventions taken. This could be due to the fact that clerical work and phone calls do not add value to the laboratory result, so removing them significantly reduced waiting times. In addition, the implementation of bar coding and the nearest sample collection room can decrease the time spent on identification, labeling, and transportation during manual data processing and apart sample collection.

Findings from the study conducted in the USA at the emergency department laboratory showed that after the intervention, lean identifies and removes non-value-added steps, including sample collection from multiple emergency departments, distraction of laboratory technicians, and clinical laboratory assistant times. Eliminating these wasteful steps leads the technologists to be focused on testing and reduces transportation time for the sample collection. After the intervention, the total TAT in chemistry and urinalysis section reduced from 127 to 82 and 174 to 67 minutes with 35.4% and 61.4% reduction, respectively. However, the total TAT for microbiology rapid tests didn’t change [36]. This might be due to the laboratory staff strictly following the standard SOP and completing the process with the targeted TAT. In addition, the discrepancy could be due to the fact that since rapid tests were performed with kits and/or strips, they didn’t require any extra activities that waste processing time.

A prospective interventional study conducted at the core laboratory in Chile demonstrated that applying lean methodologies reduces total TAT in laboratory processes. The study revealed a reduction in TAT from 84 to 73 minutes for glucose and from 54 to 53 minutes for hematocrit marking 13% and 1.8% decrease, respectively. The majority of the identified inefficiencies were related to laboratory staff functions, workflow, and infrastructure. To address these issues, the study implemented key interventions such as process redesign, redistribution of laboratory staff functions, and prioritization of activities [19]. These measures involved assigning specific tasks to each laboratory staff member and reorganizing the laboratory process and workflow based on the relevance and urgency of activities. As a result, the study eliminated 11 minutes of non-value-added time, leading to increased patient satisfaction and improved quality of service.

The study conducted in a microbiology laboratory in Spain highlighted improvements in laboratory TAT for HIV rapid testing and COVID-19 tests. For HIV rapid testing, the TAT was reduced dramatically from 2002 minutes to 251 minutes, representing an 87.4% reduction. Similarly, the TAT for COVID-19 tests was reduced from 624 minutes to 504 minutes, marking a 19.2% reduction. The study identified various manual activities, such as sample registration, identification, separation, re-rocking, and tube uncapping, as non-value-added activities that prolonged TAT unnecessarily. By eliminating the 24-hour refrigeration of samples, optimizing workflows, and automating these manual activities, the laboratory achieved significant reductions in TAT [37]. This strategic change, along with the adoption of automated and faster equipment, not only cut down the TAT but also enhanced patient satisfaction and the overall quality of service.

As evident from the literature reviewed, the integration of lean methodology into pathology laboratory operations has shown a reduction in laboratory TAT, with reported reductions ranging from 4 [34] to 6137 [10] minutes. However, these variations can be attributed to several factors such as differences in sample size, study duration, and the specific intervention measures implemented. Moreover, given the diverse range of testing procedures within pathology laboratories, including biopsies and rapid tests, there is inherent variability in the activities performed, leading to fluctuations in the total TAT reduction achieved. Conversely, in emergency laboratories, a reduction in overall TAT by 12 [19] to 152 [36] minutes has been observed. Again, these disparities can be attributed to factors such as the scope of testing conducted in the respective studies. The differences in the number and types of tests conducted significantly influence the outcomes of TAT reduction initiatives.

In summary, the variance in TAT reduction across studies can be attributed to multiple factors, including the specific study setting, design, departmental focus, types of tests conducted, sample sizes, and the effectiveness of intervention measures implemented. Since turnaround time and customer satisfaction were the major quality indicators of the laboratory, lean played a great role in reducing laboratory TAT and increasing patient satisfaction through the identification of wasteful procedures. The implementation of lean aimed to identify non-value-added steps, resulting in different intervention measures to be taken. As a result, every laboratory should pay attention to lean and integrate it into the routine process for better quality and continuous improvement. Identifying and eliminating wastes is crucial for improving efficiency and delivering greater value to the customer.

### Challenges on the implementation of lean

Integrating lean methodology into each laboratory operation is not so easy. There are different challenges identified from different literatures for the implementation of lean. The major challenge was resistance of laboratory’s staffs against lean methodology and difficulty of convincing them to adhere and maintain to the changes [34, 43, 44]. Another challenge was lack of laboratory information system integration and voice recognition system because it requires involvement of institutional management board and experted professionals which takes long time [10].

There are also difficulties related to inventory management; because of seasonal volume changes and costly of batch and lot purchasing, instrument reagent supplies were not instituted [36]. The review also demonstrates that the biggest obstacle to the implementation of lean concepts in medical laboratories is a lack of management support. It was also found that a lack of Lean culture in the laboratory and a lack of expertise and conceptual understanding contributed to part of the resistance to the use of Lean concepts in medical laboratory services [44].

### Limitations

The reviewed studies have several limitations. First, the small sample size, due to the limited number of published articles, restricts the generalizability of the results to the broader population. Second, inconsistencies in the definitions and measurement of the outcome of interest across the studies hindered the ability to perform a meta-analysis.

## Conclusion and recommendation

The review assured that the integration and implementation of lean into routine laboratory testing are essential to creating value for customers by reducing their waiting times. Implementing lean principles is crucial for pinpointing and removing inefficient processes. By applying these techniques, laboratories can significantly reduce turnaround times and enhance customer satisfactions. It also increases the overall clinical laboratory quality and is vital for continuous quality improvement. To enhance efficiency and quality in clinical laboratories, it is recommended that all clinical laboratories adopt and implement lean principles in their routine testing processes. The medical laboratory industry should provide ongoing training, and creates awareness for laboratory professionals to use lean as a routine activity in the laboratory medicine. Employees should actively participate in trainings and should practice lean techniques to ensure successful implementation.

## Supporting information

S1 Table Searching strategy for the impact of lean on clinical laboratory turnaround time. (DOCX)

S2 Table Summary characteristics of articles included in the systematic review (N = 7). (DOCX)

S3 Table Johanna Briggs Institute (JBI) quality assessment and critical appraisal checklist (N = 7). (DOCX)

S4 Table PRISMA 2020 checklist. (DOCX)

S5 Table List of studies excluded from the review. (DOCX)
