# Supplementary material for: Improving laboratory turnaround times in clinical settings: A systematic review of the impact of lean methodology application
Source: PLoS One. 2024 Oct 17;19(10):e0312033. doi: 10.1371/journal.pone.0312033 (PMC11486360; doi:10.1371/journal.pone.0312033)
Supplement: S1 Table — (DOCX) [file pone.0312033.s001.docx]

**S1 Table:** Searching strategy for the impact of lean on clinical laboratory turnaround time

| **Data bases** | **N/o** | **Terms** | **Hits** |
| --- | --- | --- | --- |
| **PubMed** | #1 | (((lean[Text Word]) OR ("lean methodology"[Text Word])) OR ("lean principle"[Text Word])) OR ("Toyota production system"[Text Word]) | 44,048 |
|  | #2 | ((("laboratory turnaround time"[Text Word]) OR ("laboratory TAT"[Text Word])) OR ("turnaround time"[Text Word])) OR (TAT[Text Word]) AND (fft[Filter]) | 20,100 |
|  | #3 | "clinical laboratory"[Text Word] AND (fft[Filter]) AND (fft[Filter]) | 36,864 |
|  | #1 &#2&#3 | ((((((((lean[Text Word]) OR ("lean methodology"[Text Word])) OR ("lean principle"[Text Word])) OR ("Toyota production system"[Text Word])) AND ("Laboratory turnaround time"[Text Word])) OR ("Laboratory TAT"[Text Word])) OR ("turnaround time"[Text Word])) OR (TAT[Text Word])) AND ("Clinical laboratory"[Text Word]) | 400 |
| **Scopus** | | lean OR "lean methodology" OR "lean principle" OR "Toyota production system" AND "laboratory turnaround time" OR "laboratory TAT" OR "turnaround time" OR tat AND "clinical laboratory" AND ( LIMIT-TO ( LANGUAGE , "English" ) ) | 243 |
| **Embase** | | ((lean OR 'lean methodology'/exp OR 'lean methodology' OR 'lean principle' OR 'toyota production system') AND 'laboratory turnaround time' OR 'laboratory tat' OR 'turnaround time'/exp OR 'turnaround time' OR tat) AND ('clinical laboratory'/exp OR 'clinical laboratory') AND [article]/lim AND [english]/lim | 608 |
| **Google scholar** | | “lean methodology” "laboratory turnaround time" | 114 |
| **Total articles screened** | | | **1003** |
