# Supplementary material for: Improving laboratory turnaround times in clinical settings: A systematic review of the impact of lean methodology application
Source: PLoS One. 2024 Oct 17;19(10):e0312033. doi: 10.1371/journal.pone.0312033 (PMC11486360; doi:10.1371/journal.pone.0312033)
Supplement: S2 Table — (DOCX) [file pone.0312033.s002.docx]

**S2 Table:** Summary characteristics of articles included in the systematic review (N = 7)

| **Authors (Year)** | **Study Area** | **Study design** | **Laboratory department** | **Type of laboratory tests** | **Sample size** | | **TAT before lean (Minute)** | **TAT after lean (Minute)** | **Change in TAT (Minute)** | **Percentage of TAT reduction (%)** | **Data Extracted by** | **Date of data extraction** |
| --- | --- | --- | --- | --- | --- | --- | --- | --- | --- | --- | --- | --- |
|  |  |  |  |  | **Before lean** | **After lean** |  |  |  |  |  |  |
| YR Mujtabai et al., (2020) | Lahore | Cross-sectional | Pathology laboratory | Overall testing | 35 | 35 | 8,022 | 1,885 | 6,137 | 76.5 | NC | Feb 6, 2024 |
| S Isa et al., (2020) | Malaysia | Cross-sectional | Pathology laboratory | Overall testing | 1,384 | 1,394 | 35 | 31 | 4 | 11.4 | ZY and MN | Feb 27, 2024 |
| Rutledge et al., | USA | Interventional | Core Pathology laboratory | Overall testing | NA | NA | 54 | 23 | 31 | 57.4 | MT | March 4. 2024 |
| Gupta S et al., (2018) | India | Experimental | Pathology laboratory | Hematology | 120 | 120 | 179.49 | 94.7 | 84.79 | 47.2 | DMB and BBT | March 2, 2024 |
|  |  |  |  | Biochemistry | 95 | 95 | 267.71 | 208 | 59.71 | 22.3 |  |  |
|  |  |  |  | **Total TAT** | **215** | **215** | **447.2** | **302.7** | **144.5** | **32.3** |  |  |
| Benjamin A. et al., (2015) | USA | Longitudinal | Emergency laboratory | Chemistry | 4,378 | 5,542 | 127 | 82 | 45 | 35.4 | EC and AMT | March 19, 2024 |
|  |  |  |  | Urinalysis | 21,162 | 44,178 | 174 | 67 | 107 | 61.4 |  |  |
|  |  |  |  | Microbiology | 1,193 | 2,436 | 37 | 37 | 0 | 0 |  |  |
|  |  |  |  | **Total TAT** | **26,733** | **52,156** | **338** | **186** | **152** | **44.9** |  |  |
| Letelier P et al., (2021 | Chile | Quasi-experimental | Emergency Laboratory | Glucose | 6,684 | 6,684 | 84 | 73 | 11 | 13.0 | NC | Feb 6, 2024 |
|  |  |  |  | HCT |  |  | 54 | 53 | 1 | 1.8 |  |  |
|  |  |  |  | **Total TAT** | | | **138** | **126** | **12** | **8.6** | AAA | March 22, 2024 |
| Acero R et al., (2023) | Spain | Longitudinal | Microbiology Laboratory | HIV | 83,660 | 83,660 | 2002 | 251 | 1751 | 87.4 |  |  |
|  |  |  |  | COVID- 19 | 136,488 | 136,488 | 624 | 504 | 120 | 19.2 |  |  |
|  |  |  |  | **Total TAT** | **220,148** | **220,148** | **2,626** | **755** | **1,871** | **71.2** |  |  |
| **Total** | | | | | **255,199** | **280,632** | **11,660.2** | **3,308.7** | **8,351.5** | **71.6** |  |  |

**Key:** TAT: Turnaround time; ED: Emergency Department; TT: Troponin Total; US: Urine Sedimentation; TI: Troponin I; UA: Urinalysis; UHCG: Urine Human Cortico Gonadotropic Hormone; MRT: Microbiology Rapid Testing; HCT: Hematocrit; HIV: Human immunodeficiency Virus; NA: Not Applicable
