# Supplementary material for: Improving laboratory turnaround times in clinical settings: A systematic review of the impact of lean methodology application
Source: PLoS One. 2024 Oct 17;19(10):e0312033. doi: 10.1371/journal.pone.0312033 (PMC11486360; doi:10.1371/journal.pone.0312033)
Supplement: S3 Table — (DOCX) [file pone.0312033.s003.docx]

**S3 Table:** Johanna Briggs Institute (JBI) quality assessment and critical appraisal checklist (N = 7)

| **Quality assessment of Interventional studies included in the review using JBI critical appraising checklist** | | | | | | | | | | | | | |
| --- | --- | --- | --- | --- | --- | --- | --- | --- | --- | --- | --- | --- | --- |
| **S/N** | **Authors** | **Q1** | **Q2** | **Q3** | **Q4** | **Q5** | **Q6** | **Q7** | **Q8** | **Q9** | **Total score** | **percent Scored** | **Remark for illegibility** |
| 1 | Roe Rutledge & Joame Simpson | Yes | Yes | NA | Yes | Yes | Yes | Yes | Yes | Yes | 8 | 88.9% |  |
| 2 | Gupta S, Kapil S, and Sharma M | Yes | Yes | Yes | Yes | Yes | Yes | Yes | Yes | Yes | 9 | 100% |  |
| 3 | Letelier P et al., | Yes | Yes | Yes | Yes | Yes | Yes | Yes | Yes | Yes | 9 | 100% |  |
| **Quality assessment of cross-sectional studies included in the review using JBI critical appraising checklist** | | | | | | | | | | | |  |  |
| **S/N** | **Authors** | **Q1** | **Q2** | **Q3** | **Q4** | **Q5** | **Q6** | **Q7** | **Q8** | **Total score** | |  |  |
| 4 | YR Mujtabai et al., | Yes | Yes | Yes | UC | Yes | Yes | Yes | Yes | 8 | | 100% |  |
| 5 | S Isa et al., | Yes | Yes | UC | Yes | Yes | Yes | Yes | Yes | 7 | | 87.5% |  |
| 6 | Acero R et al., | Yes | Yes | Yes | Yes | Yes | Yes | Yes | Yes | 8 | | 100% |  |
| 7 | Benjamin A. et al., | Yes | UC | Yes | Yes | Yes | Yes | Yes | Yes | 7 | | 87.5% |  |

**Key:** Yes: Done; No: Not done; UC: Unclear: NA: Not Applicable; √: Article illegible to be included in the review

**Questions for Interventional studies**

1. Is it clear in the study what is the “cause” and what is the “effect” (i.e., there is no confusion about which variable comes first)?
2. Was there a control group?
3. Were participants included in any comparisons similar?
4. Were the participants included in any comparisons receiving similar treatment/care, other than the exposure or intervention of interest?
5. Were there multiple measurements of the outcome, both pre and post the intervention/exposure?
6. Were the outcomes of participants included in any comparisons measured in the same way?
7. Were outcomes measured in a reliable way?
8. Was follow-up complete and if not, were differences between groups in terms of their follow-up adequately described and analyzed?
9. Was appropriate statistical analysis used?

**Questions for cross-sectional studies**

1. Were the criteria for inclusion in the sample clearly defined?
2. Were the study subjects and the setting described in detail?
3. Was the exposure measured in a valid and reliable way?
4. Were objective, standard criteria used for measurement of the condition?
5. Were confounding factors identified?
6. Were strategies to deal with confounding factors stated?
7. Were the outcomes measured in a valid and reliable way?
8. Was appropriate statistical analysis used?
