# Supplementary material for: Improving laboratory turnaround times in clinical settings: A systematic review of the impact of lean methodology application
Source: PLoS One. 2024 Oct 17;19(10):e0312033. doi: 10.1371/journal.pone.0312033 (PMC11486360; doi:10.1371/journal.pone.0312033)
Supplement: S5 Table — (DOCX) [file pone.0312033.s005.docx]

**S5 Table:** List of Excluded articles with reasons

| **Primary author (Year)** | **Reasons for exclusion** |
| --- | --- |
| Chiasakul (2018), Petersen (2019), Salam (2023), Sonagra (2024), L'Ollivier (2017), Riben (2015), Valenstein (1996), Ward  (2020), Mathew (2024), Zhang (2014), Khare (2023), Tolan (2022), Antonios (2021), Heifets (1997), Okubazgi (2020), Qavi (2023), Getawa (2022), McMullen (2016), Salinas (2016), Alikian (2012), Hawker (2017), Dolci (2017), Lippi (2014), Babady (2016), Rossen (2018), Archetti (2017), Swiner (20202), Dimenstein (2020), Frater (2022), Huf (2022), Oommen (2017), Chandler (2013), Cao (2022), Petrides (2022), Kim (2022), Sande (2020), Landsburg (2023), Li (2023), Tan (2020), Krishna (2012), Cotta (2022), Mackie (2020), Schwalm (2018), Niyonzima (2021), Qin (2020), Cao (2016), Debuysschere (2023), Shi (2021), Müller (1999), Zaninotto (2010), Landsburg (2022), Namiki (1994), Namiki (2010), Uechi (2013), Haber (1990), Okabe (2005), Xu (1998), Ceppa (2008), Goldenberg (2011), Sivaraman (2013), Wah Fong (2009), Hoppensteadt (1999), Strachan (2013), Mor (2014), Kvach (2010), Poerio (2020), Li (2008), Sheppard (2008), Clancy (1997), Hayette (2020), Schneiderhan (2013), Steffes (1996), Kanno (2000), Saeki (2001, Syrmis (2004), Steijns (2002), Weber (2003), Ismail (2004), Hilborne (1996), Ohta (2011), Crocker (2013), Mann (2010), Emmerich (1998), Yang (2010), McCollum (2014), Babady (2012), Catton (2011), Pfaller (1984), Singh (2011), Barko (2006), Roubille (2010), Hofgärtner (1999), Marshall (2001), Binnicker (2010), Wu (2014), Ratnam (1995), Cherkaoui (2010), Walkty (2013), Jayaratne (2013), Şamlı (2016), Khan (2008), Sakamaki (2012), Glencross (2014), Koehler (1999), Kim (2018), Mitra (2020), Quinlan (2015), Wang (2008), Yao (2010), Frans (2019), Burchard (2013), Huang (2013), Georgiou (2014), Espy (2000), Hänscheid (2003), Guo (2015), Horowitz (2005), Owens (2010), Gardam (2001), Volmar (2013), Bruins (2011), Gao (2012), Tóth (2014), Park (2016), Roosevelt (2001), Manickam (2013), Migliavacca (2024), Georgiou (2011), Vanstone (2018), Barenfanger (2002), Chan (2002), Nkrumah (2011), Jones (2006), Westbrook (2009), Park (2013), Wlazeł (2015), Deetz (2013), Porter (2020), Fanshawe (2018), Wolters (2020), Maurice (2010), Deetz (2012), Zimmermann (2011), Hefetz (2024), Basu (2020), Singh (2024), Jian (2012), Novis (2004), Agurto (2006), You (2013), Toulon (2009), Wölfel (2015), Chan (2021), Di Serio(2005), Dainiak (2007), Lippi (2017), Salem (1991), Meier (2015), Lucic (2013), Jaso (2011), Isaksson (2018), Balbás (2017), Burchard (2014), Tucker (2024), Broeren (2011), Wang (2017), Beadling (2013), Abou (2015), Vakili (2020), Granato (2018), Duan (2018), Morey (2016), Janssens (2014), Almeida (2009), Ambachew (2018), Friedman (1993), Addis (2013), Mehrotra (2017), Holland (2006), Paugam (2013), Di Serio (2006), Bali (2015), Saxena (1993), Koçman (2018), Rimac (2020), Yu M(2019), Marson (2017), Sarzotti-Kelsoe (2014), Navidad  (2013), Caruso (2023), Steindel (1999), Valenstein (2003), Roubille (2010), Akubulut (2023), Song (2018),,,,,,,,(N=949) | Articles excluded by titles and abstract (didn’t meet the eligibility criteria) |
| Lagamayo (2023), Kumar (2022), Hagg (2007), Lokesh (2020), Agarwal (2015), Tamer (2017), Niketa (2022), Yasang (2016), Inal (2018), Lou (2017), Alain (2021), Badrick (2021), Yao (2010), Westbrook (2009), Viccellio (2008), Wu AHB (2019), Bhatt (2019), Burke (1997), Ehlers (2018), Vrijsen (2022), Lam (2012), Park (2023), Khalifa (2014), Zhang (2018), Cankovic (2009), Shiferaw (2019), Gjolaj (2014), Jones (2022), Prijatelj (1999), Behling (2015), Holland (2005), Pelegrí (1996), Ialongo (2016), Stotler (2012), Wu (2018), Tornel (2005), Fei (2015), Lee-Lewandrowski (2003), Okorodudu (2009), Mutingi M (2017), Moore (2019), Antal-Szalmás (2007), Chung (2009), Kilgore (1998) (N=44) | Articles not reporting the outcome of interest |
| Hilma (2017), Diana C (2019), Kumar (2022) (N=3) | Articles with ambiguous sample size and result |
